# Comparison of health access, lifestyle, prostate cancer knowledge and screening among black men residing in West Africa and the USA

**DOI:** 10.3332/ecancer.2021.1309

**Published:** 2021-10-25

**Authors:** Orlando Rivera Colón, Opeyemi Bolajoko, Folakemi Odedina, Folakemi Odedina

**Affiliations:** 1 School of Kinesiology and Physical Therapy, Department of Health Sciences, University of Central Florida, Orlando, Florida, USA; 2 Nutrition and Dietetics Department, Federal University of Agriculture, PMB 2240, Abeokuta, Ogun State, Nigeria; 3 Department of Pharmacy, University of Florida, Gainesville, Florida, USA; * Prostate Cancer Transatlantic Consortium

**Keywords:** prostate cancer, health access, screening, black men, insurance coverage

## Abstract

**Background:**

In Blacks, late presentation, lack of knowledge, health infrastructural deficiencies and socio-demographic characteristics, which result in poor outcomes, are the bane of cancers. This study evaluated health access and lifestyle association with prostate cancer (PCa) knowledge and screening among black men.

**Methodology:**

This study used data from the Prostate Cancer Transatlantic Consortium familial cohort study. Data were gathered from a cross-sectional survey of 500 community-dwelling black men in Nigeria, Cameroon, and the USA. Information on socio-demographics, health care access, PCa knowledge score and screening behaviour was obtained, and the association between these variables was evaluated.

**Results:**

The majority (81.6%) were Nigerian. The age ranges were 35–49 (55.2%) and ≥65 (8.4%). The income distribution of the respondents showed that 23.3% earned <$1,000 and 30.7% (>$2,000) monthly. Only 43% had health insurance coverage, and 12% had accessed a doctor in 12 months. Respondents relied on orthodox medicine (50.8%), neighbourhood pharmacy (10.6%), self-medication (5%) and neighbourhood nurse (24.6%). The participants had either poor (45.2%) or very poor (23.2%) dietary patterns. Most (66.67%) do not engage in physical activity and about 33.33% engage in some exercises. Moreover, 87.8% and 78.3% have never had a digital rectal examination (DRE) and prostate-specific antigen (PSA) screening in their lifetime, respectively, while 6.8% and 1.6% had DRE last 1 year and 2 years, respectively. Furthermore, 65.2%, 19.8% and 15% of the respondents had poor, fair and good knowledge of PCa, respectively. Health care coverage (*p* < 0.001), medical care habit (p = 0.001), routine checkup (*p* = 0.013) were significantly associated with respondents’ PCa knowledge. Routine checkup (*p* < 0.001) and country (*p* < 0.001) were significantly related to PSA screening.

**Conclusion:**

The study showed that PCa screening uptake was poor among the respondents and country of residence was associated with PCa screening behaviours. Healthcare coverage was significantly associated with PCa knowledge.

## Introduction

Prostate cancer (PCa) is a public health concern among Black men. Studies have shown a 60% higher incidence among black men than in whites [1]. However, easy access to health care services, appropriate changes in some behavioural lifestyles, such as physical activity (PA) and good dietary patterns could be a key component for preventing or slowing the progression and improving PCa prognosis [2]. Some studies showed that PA (moderate or vigorous) is associated with slowing down PCa progression [2, 3]. It is still an understudied field; more studies on PA and cancer prevention are required [4]. In addition, dietary pattern has the potential to reduce the risk of PCa and its progression. It was reported that lowering the intake of energy-dense foods and increasing the variety of vegetables, fruits, whole grains and pulses consumed as well as the sparing use of red and processed meats could reduce the risk of chronic diseases, including PCa [3].

Access to health care refers to the ease with which an individual can obtain needed medical services. In Africa, poor health care access is the cause of morbidity and mortality from non-communicable chronic diseases, such as cancer. This high mortality could have resulted from the late presentation and diagnosis of patients in Africa predicated on poor health care access observed in countries in sub-Saharan Africa. Health care access could be an issue among Blacks, preventing them from participating in PCa screening. In addition, poverty results in inappropriate patients’ health-seeking behaviours since most health care bills are borne by the patients and caregivers who make out-of-pocket payments [5, 6], due to inadequate and lack of access to health insurance services by many sub-Saharan Africa residents. The shortage of health professionals evident by the health worker per thousand-population ratio in Africa’s health care system, especially Nigeria, is appalling and attests to this [7–9]. The long patient queue to see clinicians clearly shows the accessibility of health services to patients and their caregivers [10].

Conversely, the PCa knowledge and screening could induce behavioural change, as reported by previous studies [11, 12]. Those with PCa knowledge were found to be more favourably disposed to participate in screening [13]. However, knowledge may not necessarily lead to behavioural changes like PA and dietary patterns [14]. It is not yet established that knowledge of the disease would change PA and dietary patterns. Although if proper education and recommendation are based on specific procedures and models, change towards PA and diet might be achieved.

On the other hand, PCa is the most common cancer among men of African descent in North Americans and Africans [15]. However, knowledge and screening behaviours could differ across countries, translating to the incidence and prevalence of PCa with higher figures attributed to the developed countries. This high prevalence can be attributed to better health insurance coverage and health care access in developed countries [16]. In North America, an incidence of 73.7 per 100,000 population, followed by Europe (62.1), was reported. In contrast, Africa and Asia have lower incidence rates of 26.6 and 11.5, respectively, compared to the developed countries [15, 17]. The difference in variables might be due to variations in screening between developed and developing countries. Studies have suggested poor screening uptake in developing countries [18]; this could be responsible for the low incidence and prevalence [17]. For example, the USA is the second in accounting for cancer (9.5%) of 164,690 PCa cases registered in 2018 [19]. While Europe is number one, accounting 24% of all new cancer (450,00 PCa cases) reported in 2018 [17].

Thus, this study aimed to assess health care access and knowledge of Pca associated with screening behaviour and lifestyle factors, including moderate-intense exercise and dietary patterns among black men in Nigerian, Cameroon, and US populations. From the objective of the study, there were two hypotheses. First, there is more knowledge of PCa among the U.S. community-dwelling men than in Nigeria and Cameroon. Second, PCa screening behaviour is associated with health access, moderate-intense exercise and good dietary pattern.

## Methods

This study was a retrospective analysis that used the cross-sectional survey of the Prostate Cancer Transatlantic Consortium (CaPTC) familial cohort, carried out among 500 community-dwelling male respondents between 35 and 70 years old. The data collection method has been previously published [20]. The socio-demographic characteristics, health care access, PCa knowledge and screening behaviours of respondents were assessed. In addition, respondents’ physical activities and dietary patterns were evaluated. Respondents were asked if they had access to a medical doctor in the last 12 months, go for a routine health checkup. They were also asked to state how medical bills were paid and their first response to medical challenges. Moreover, respondents were asked if they engaged in a vigorous-intensity activity that increases their breathing or heart rate, such as lifting heaving loads, digging or construction work. They were further asked the frequency of these physical activities per week. The answer option was a Yes and No for the first question and they stated the number of days per week for the second question on physical activities. The same question model was repeated for moderate-intensity exercises, such as activities that cause a small increase in breathing or heart rate, such as brisk walking. Furthermore, we evaluated how regularly participants exercised in their free time, ranging from 0 to 7 days in a week. Knowledge of PCa was assessed using a 20 points scale, which was scored and categorised. Poor knowledge was categorised as a score of 0%–39%, while 40%–69% was moderate knowledge and ≥70% as good.

The dietary pattern of the participants was assessed using a food frequency questionnaire, in which they were asked to indicate how often and the forms in which foods and drinks were taken per week. To calculate the food variety score, we adapted the scoring procedure and categories of the food variety checklist by Keflie *et al* [21]. A score of 1 was given for each food item eaten. The aggregate food variety score was totalled and categorised as ≥54% very good, 45%–53% was good, 36%–44% fair, 18%–35% poor and <18% was very poor. Data were analysed for descriptive and inferential statistics using SPSS version 20. Continuous variables were presented as means and standard deviation, while categorical variables were presented as frequency counts and percentages. Associations between categorical variables were determined using chi-square, while variations in numeric variables were determined using analysis of variance. The significant level for all variables was set at *p*-values < 0.05.

## Results

Table 1 provides socio-demographic characteristics of 500 participants, of which 84.4% were from Nigeria, 6.0% lived in Cameroon and 7.2% lived in the USA. The majority (55.2%) of the respondents were 35–49 years old, 28.4% had postgraduate education and 21.2% had an income of 1,000,000 naira and above. Employment status showed that 50.8% were employed for wages while 22.6% were self-employed (Table 1). The occupation distribution of the respondents showed that 29.0% were professionals while 4.2% were farmers (Table 1). Location (<0.001), age (0.019), education level (<0.001), income (<0.001) and occupation (<0.001) were significantly associated with respondents’ PCa knowledge.

Respondents’ PCa knowledge showed that 15.0%, 19.8% and 65.2% of the respondents had good, fair and poor knowledge, respectively. The participants had either poor (45.2%) or very poor (23.2%) dietary patterns. Most (64.8%) respondents do not engage in PA and about 32.4% engage in some types of activity. Majority of the respondents have never undergone prostate-specific antigen (PSA) (75.6%) and digital rectal examination (DRE) (84.4%) tests (Table 2). Table 3 showed the correlation between variables and screening uptake among the respondents. There was a strong correlation between PSA and DRE screening (*r* = 0.638). There is no correlation between PSA and DRE screening and dietary pattern (*r* = −0.054 and −0.049, respectively), and as knowledge of PCa increases, the quality of dietary pattern decreases (*r* =−0.142).

Table 4 provides information on health care access of the respondents, only 33.2% had health insurance cover, and 10% had accessed a doctor in the past 12 months. For a routine checkup, 60.4% never had a routine medical checkup, while 18.8% have done medical checks once in the last 12 months, 52.6% personally pay for their healthcare cost, 6.2% had their medical bills paid by insurance and 12.8% received government subsidy. Respondents’ healthcare habits show that 42.4% relied on orthodox medicine for medical care, neighbourhood pharmacy (9.4%), self-medication (4%) and neighbourhood nurse (20.4%). Health care coverage (*p* < 0.001), medical care habit (*p* = 0.001), routine checkup (*p* = 0.013) were significantly associated with respondents’ PCa knowledge. Moreover, Health care coverage (*p* < 0.043), routine check-up (*p* < 0.001) and access to a doctor in the past 12 months (*p* < 0.001) were found to be significantly related to the country of residence.

Table 5 presents the comparison between PCa screening, PA and dietary patterns. Nigeria has the worst screening behaviour as 82.1% and 90.5% never had PSA and DRE screening. More proportion (51.6%) of Cameroonians have had PSA screening than Nigerian and US black men. However, more proportion (31.7%) of the respondents from the USA have had DRE screening. Country of residence was found to be significantly associated with PCa knowledge (*p* = 0.015), PSA (*p* ≤ 0.001) and DRE (*p* ≤ 0.001) screening, but not with dietary pattern (*p* = 0.116).

## Discussion

This study assessed the knowledge and screening behaviour of community-dwelling Blacks as well as their health care access, physical activity and dietary pattern. The study found poor health care access and screening behaviour in that majority have never screened for PSA or DRE. In addition, poor dietary pattern was observed in a majority of respondents. As knowledge of PCa increases, the quality of dietary patterns decreases (*r* = −0.142). African Americans tend to have more DRE screening than their West African counterparts.

Many studies have identified factors associated with PCa screening, including the perceived invasive nature of DRE that might be against their cultural beliefs, as observed by James *et al* [22]. Their study across eight countries found that preserving masculinity (bodily invasion, losing sexuality, threatening manhood and medical avoidance) was the reason for not screening. In this study, more participants took PSA testing than DRE, which might be due to the invasive nature of DRE test. Moreover, the poor screening behaviour observed in this study might be due to poor health access and low income because most spending on health is usually out of pocket and may not be affordable [23, 24]. An earlier study by Kaninjing *et al* [25] found that income, health access and cultural belief could be why many African men were not screened. In addition, due to poor uptake of health insurance services in Africa, health access tends to be mainly poor, and many might not access necessary health information that might encourage screening [24, 26].

There is ample evidence suggesting that PA and exercise can be therapeutic tools for PCa patients. Also, it has been found to prevent chronic diseases, such as cancer [27, 28]. However, in this study, more than half of the participants did not engage in physical activities. A study showed that participants had improved their knowledge in exercise, but there was no significant PA engagement increase [29]. This suggests that knowledge might not necessarily lead to practice.

More than half of the participants had poor dietary patterns. However, PCa screening behaviour has no association with PA and dietary patterns. Poor dietary patterns and sedentary lifestyles have been implicated in the onset of chronic diseases [4, 30]. A previous study associated all invasive cancer with poor/suboptimal diet [31]. Another study demonstrated a relationship between lack of health insurance and a high risk of chronic diseases and poor diet [32]. In addition, a study from southwest Nigeria assimilated large households with poor dietary intake as well they demonstrated a secondary level of education [33]. They implied that a high level of knowledge could positively impact and influence their nutritional uptake. These also corroborated with the results found in PCa knowledge, in which 65.2% had poor PCa knowledge, which is lower than the 86.1% poor knowledge among retired men [34]. As PCa knowledge increases, dietary pattern decreases; it could be because there might not be a specific dietary guideline for PCa. Studies have demonstrated that PCa knowledge does not necessarily mean screening and knowledge was not found to predict screening behaviour [35, 36].

Other studies from Nigeria showed that although knowledge and screening were low, those with good PCa knowledge were willing to screen more than those with poor knowledge [37]. The fact that the cost of screening was high was one of the reasons why they could not screen and lack of health insurance. Only age and location were associated with screening. As participants get older, they tend to screen more, which might be due to health challenges and doctor prescriptions. Location association might be because of poor health access and income. As a study demonstrated, older participants engage in screening because of their health insurance coverage and physician suggestions. Although, more white men (33%) than black men (25%) screened more due to differences in socio-economics level [31, 38]. Lastly, as mentioned by Taitt [38], locality was found to be significantly associated with PCa screening due to various factors like genes, socio-economic and health care access.

The study has limitations. First, the study evaluated secondary data; therefore, causal relationships cannot be confirmed. The data were from community-dwelling men in Nigeria and Cameroon with relatives in the USA; thus, the outcome might not be generalisable for blacks in the USA. Wide variations in demographics and the health care system might have impacted this study. Moreover, most of the respondents were recruited from Nigeria, making the data skewed. This might have affected the outcomes of this study. There is a need for more prospective studies of men with PCas or disorders to validate the outcomes of this study.

## Conclusion

The study showed that healthcare access and screening uptake were poor among the respondents, and socio-demographic characteristics were not associated with PCa screening behaviours. There was a significant association between screening behaviour across the countries and healthcare access was significantly associated with PCa knowledge.

## Conflicts of interest

There was no conflicts of interest.

## Figures and Tables

**Table 1.** Education and income status.

	Frequency	Percent	*p*-values		
			PCa knowledge	PSA screening	DRE screening
**Location**			0.015	<0.001	<0.001
Nigeria	422	84.4			
Cameroon	30	6.0			
USA	38	7.6			
Non-response	10	2.0			
**Age (years)**			0.019	<0.001	<0.001
35–49	276	55.2			
50–64	182	36.4			
65 and above	42	8.4			
**Education**			<0.001	0.825	0.202
Primary	2	0.4			
Secondary	37	7.4			
High	71	14.2			
Technical	24	4.8			
University	39	7.8			
Postgraduate	142	28.4			
Refused	101	20.2			
Non-response	84	16.8			
**Employment status**					
Employed for wages	254	50.8			
Self-employed	113	22.6			
Out of work for more than 1 year	8	1.6			
Homemaker	1	0.2			
Student	2	0.4			
Retired	37	7.4			
Unable to work	2	0.4			
Non-response	83	16.6			
**Income**			<0.001	0.054	0.050
<100,000	43	8.6			
100,000–199,999	46	9.2			
200,000–299,999	21	4.2			
300,000–399,999	21	4.2			
400,000–499,999	14	2.8			
500,000–599,999	20	4.0			
600,000–699,999	16	3.2			
700,000–799,999	10	2.0			
800,000–899,999	14	2.8			
900,000–999,999	13	2.6			
1,000,000 and above	106	21.2			
Refused	92	18.4			
Non-response	84	16.8			
**Occupation**			<0.001	0.976	0.996
Professional	145	29.0			
Managerial	36	7.2			
Technical	64	12.8			
Operator/fabricators/factory	13	2.6			
Service	45	9.0			
Farmer	21	4.2			
Artisan	29	5.8			
Others	55	11.0			
Refused	2	0.4			
Non-response	92	18.4			

**Table 2.** Lifestyles and screening behaviours of respondents.

	*N*	%
Physical activity		
No	324	64.8
Yes	162	32.4
Non-response	14	2.8
Dietary pattern		
Very good	40	8
Good	41	8.2
Fair	77	15.4
Poor	226	45.2
Very poor	116	23.2
PCa knowledge		
Good	75	15
Fair	99	19.8
Poor	326	65.2
PSA		
Never	378	75.6
Past year	70	14.0
Past 2 years	18	3.6
Past 3 years	9	1.8
Past 4 years	2	0.4
5 or more years ago	6	1.2
Non-response	17	3.4
DRE		
Never	422	84.4
Past year	34	6.8
Past 2 years	8	1.6
Past 3 years	4	0.8
Past 4 years	6	1.2
5 or more years ago	9	1.8
Non-response	17	3.4

**Table 3.** Correlation between dietary pattern, PCa knowledge and screening.

Variables	Dietary pattern	Age group	PSA	DRE	PCa knowledge
Dietary pattern	1.000	−0.138**	−0.054	−0.049	−0.142**
Age group		1.000	0.369**	0.306**	0.019
PSA			1.000	0.638**	−0.113*
DRE				1.000	−0.035
PCa knowledge					1.000

* Significant at 0.05,

** Significant at 0.001

**Table 4.** Health access and habits of respondents.

	Frequency	Percent	PCa knowledge	Country
Health care coverage			<0.001	0.043
No	234	46.8		
Yes	181	36.2		
Don’t know/not sure	3	0.6		
Refused	2	0.4		
Non-response	80	16		
Health care payment			0.093	0.308
Self	263	52.6		
Family and friends	12	2.4		
Medical insurance	31	6.2		
Government subsidy	64	12.8		
Charity	31	6.2		
Non-response	99	19.8		
Access to doctor/last 12 months			0.426	<0.001
Yes	50	10.0		
No	352	70.4		
Don’t know	9	1.8		
None response	89	17.8		
Routine check-up			0.013	<0.001
No	302	60.4		
Yes	94	18.8		
None response	104	20.8		
Medical care habit			<0.001	0.997
Nothing	4	0.8		
Pray to God	20	4.0		
Traditional/native doctor	6	1.2		
Self-medication	20	4.0		
Local pharmacy/pharmacist	47	9.4		
Nurse/physician assistant	102	20.4		
Western/medical/orthodox medicine	212	42.4		
None response	89	17.8		

**Table 5.** Comparison between respondents country of residence, screening, knowledge and dietary pattern.

	Nigeria (*N*%)	Cameroon (*N*%)	USA (*N*%)	Significance
**PSA screening**				<0.001
Never	335 (82.1%)	14 (48.3%)	23 (60.5%)	
Screened	73 (17.8%)	15 (51.6%)	15 (39.4%)	
**DRE screening**				<0.001
Never	382 (90.5%)	23 (76.7%)	26 (68.4%)	
Screened	40 (9.5%)	7 (23.3%)	12 (31.7%)	
**PCa knowledge**				0.015
Good	59 (14.0%)	5 (16.7%)	11 (28.9%)	
Fair	84 (19.9%)	5 (16.7%)	8 (21.1%)	
Poor	279 (66.1%)	20 (66.7%)	19 (50.0%)	
**Dietary pattern**				0.116
Very good	30 (7.1)	2 (6.7)	6 (15.8)	
Good	30 (7.1)	3 (10.0)	6 (15.8)	
Fair	66 (15.6)	5 (16.7)	6 (15.8)	
Poor	193 (45.7)	12 (40.0)	18 (47.4)	
Very poor	103 (24.4)	8 (26.7)	2 (5.3)	

## References

[1] Tsodikov A, Gulati R, Carvalho TM (2018). Is prostate cancer different among black men, answers from three natural history models. Cancer.

[2] Kruk J, Aboul-Enein H (2016). What are the links of prostate cancer with physical activity and nutrition? A systematic review article. Iran J Public Health.

[3] Hackshaw-McGeagh LE, Perry RE, Leach VA (2015). A systematic review of dietary, nutritional, and physical activity interventions for the prevention of prostate cancer progression and mortality. Cancer Causes Control.

[4] Peisch SF, Van Blarigan EL, Chan JM (2017). Prostate cancer progression and mortality: a review of diet and lifestyle factors. World J Urol.

[5] Manzambi Kuwekita J, Gosset C, Guillaume M (2015). Combining microcredit, microinsurance, and the provision of health care can improve access to quality care in urban areas of Africa: results of an experiment in the Bandalungwa health zone in Kinshasa, the Congo. Med Sante Trop.

[6] Vergunst R, Swartz L, Hem KG (2019). The perceived needs-access gap for health services among persons with disabilities in a rural area within South Africa. Disabil Rehabil.

[7] Schultz C, Rijks B (2014). Mobility of Health Professionals to, from and within the European Union.

[8] Of M, Workers H (2015). Migration of Health Workers.

[9] Labonté R, Sanders D, Mathole T (2015). Health worker migration from South Africa: causes, consequences and policy responses. Hum Resour Health.

[10] Aslan DI (2015). Applications of queues in hospitals in Istanbul. J Soc Sci.

[11] Koitsalu M, Eklund M, Adolfsson J (2018). Predictors of participation in risk-based prostate cancer screening. PLoS One.

[12] Akinremi TO, Adeniyi A, Olutunde A (2014). Need for and relevance of prostate cancer screening in Nigeria. Ecancermedicalscience.

[13] Ogunsanya ME, Brown CM, Odedina FT (2017). Knowledge of prostate cancer and screening among young multiethnic black men. Am J Mens Health.

[14] Knowledge PC (2017). Behaviors in jamaican men.

[15] Rebbeck TR, Devesa SS, Chang BL (2013). Global patterns of prostate cancer incidence, aggressiveness, and mortality in men of african descent. Prostate Cancer.

[16] Feng RM, Zong YN, Cao SM (2019). Current cancer situation in China: good or bad news from the 2018 Global Cancer Statistics?. Cancer Commun (London, England).

[17] Rawla P (2019). Epidemiology of prostate cancer. World J Oncol.

[18] Bello JO, Buhari T, Mohammed TO (2019). Determinants of prostate specific antigen screening test uptake in an urban community in North-Central Nigeria. Afr Health Sci.

[19] Bray F, Ferlay J, Soerjomataram I (2018). Global cancer statistics 2018: GLOBOCAN estimates of incidence and mortality worldwide for 36 cancers in 185 countries. CA Cancer J Clin.

[20] Oladoyinbo CA, Akinbule OO, Sobo AA (2018). Behavioural risk factors associated with prostate cancer: the prostate cancer transatlantic consortium (CaPTC) cohort study. J Glob Oncol.

[21] Keflie TS, Samuel A, Lambert C (2018). Dietary patterns and risk of micronutrient deficiencies: their implication for nutritional intervention in Ethiopia. J Nutrit Health Food Sci.

[22] James LJ, Wong G, Craig JC (2017). Men’s perspectives of prostate cancer screening: a systematic review of qualitative studies. PLoS One.

[23] Akinyemiju TF, McDonald JA, Lantz PM (2015). Health care access dimensions and cervical cancer screening in South Africa: analysis of the world health survey. BMC Public Health.

[24] Akpomuvie O (2010). Poverty, access to health care services and human capital development in Nigeria. African Res Rev.

[25] Kaninjing E, Lopez I, Nguyen J (2018). Prostate cancer screening perception, beliefs, and practices among men in Bamenda, Cameroon. Am J Mens Health.

[26] Ogunbiyi OJ (2011). Impact of health system challenges on prostate cancer control: health care experiences in Nigeria. Infect Agent Cancer.

[27] Stout NL, Baima J, Swisher AK (2017). A systematic review of exercise systematic reviews in the cancer literature (2005–2017). PM R.

[28] Segal R, Zwaal C, Green E (2017). Exercise for people with cancer: a systematic review. Curr Oncol.

[29] Morrow JR, Krzewinski-Malone JA, Jackson AW (2004). American adults’ knowledge of exercise recommendations. Res Q Exerc Sport.

[30] Shi L, Morrison JA, Wiecha J (2011). Healthy lifestyle factors associated with reduced cardiometabolic risk. Br J Nutr.

[31] Zhang FF, Cudhea F, Shan Z (2019). Preventable cancer burden associated with poor diet in the United States. JNCI Cancer Spectr.

[32] Bittoni MA, Wexler R, Spees CK (2015). Lack of private health insurance is associated with higher mortality from cancer and other chronic diseases, poor diet quality, and inflammatory biomarkers in the United States. Prev Med (Baltim).

[33] Omonona BT, Obisesan AA, Aromolaran OA (2015). Health-care access and utilization among rural households in Nigeria. J Dev Agric Econ.

[34] Ghodsbin F, Zare M, Jahanbin I (2014). A survey of the knowledge and beliefs of retired men about prostate cancer screening based on health belief model. Int J Community Based Nurs Midwifery.

[35] Pendleton J, Hopkins C, Anai S (2008). Prostate cancer knowledge and screening attitudes of inner-city men. J Cancer Educ.

[36] Igbokwe MC, Salako AA, Badmus T (2018). Prostate cancer and prostate cancer screening: knowledge and attitude among a semiurban population of Nigerian men. J Glob Oncol.

[37] Ukoli FA, Patel K, Hargreaves M (2013). A tailored prostate cancer education intervention for low-income African Americans: impact on knowledge and screening. J Health Care Poor Underserved.

[38] Taitt HE Global tends and prostate cancer: a review of incidence, detection, and mortality as influenced by race, ethnicity, and geographic location. Am J Mens Health.

